# ACADEMIC–PRACTICE PARTNERSHIPS IN NURSING HOMES: INTERNATIONAL EXPERIENCES, PROCESSES, AND OUTCOMES

**DOI:** 10.1093/geroni/igae098.0711

**Published:** 2024-12-31

**Authors:** Marie Boltz, Karin Wolf-Ostermann, Karen Spilsbury

**Affiliations:** Chair; Co-Chair; Discussant

## Abstract

The Academic-Practice Partnership model is increasingly gaining attention as a feasible approach to grow the geriatric workforce, generate meaningful research, and integrate evidence-based, person centered care into nursing home operations. They offer practicable approaches to optimize knowledge and skill development for students, support lifelong learning of staff, optimize research capacity and productivity, and improve resident outcomes. This symposium will describe interdisciplinary collaboration between scientists, care providers, and educators in nursing homes in three countries: United States, Germany, and the Netherlands. The engagement and experiences of stakeholders, daily operations, and research activity will be discussed. The first presentation will describe the TCALL project in Germany, which creates a direct pathway for research to care delivery via training and vice versa. The Teaching Home project in the US will be presented next, including the process of co-designing the program with nursing home leadership and staff, developing the curriculum, preparing faculty, and the content for practice. A qualitative exploration of the US students’ experiences and lessons learned in the teaching home program will be shared in the third presentation. The fourth presentation will provide an overview of the Dutch Living Lab in Long-term care; the outcomes related to quality of life of older people and their families, quality of care and quality of work will be discussed. A discussion will follow addressing future directions including inter-university collaboration, measurement considerations, and policy implications.

